# Differences in children’s exposure to television advertising of unhealthy foods and beverages in Spain by socio-economic level

**DOI:** 10.1186/s12889-023-17410-z

**Published:** 2024-03-07

**Authors:** Miguel Ángel Royo-Bordonada, Cristina Cavero-Esponera, María Mar Romero-Fernández, Cristina González-Díaz, Elena Ordaz Castillo

**Affiliations:** 1 National School of Public Health, Institute of Health Carlos III, Ministry of Science and Innovation, Madrid, 28029 Spain; 2 Department of Preventive Medicine and Public Health, Hospital Central de la Cruz Roja San José y Santa Adela, Madrid, 28003 Spain; 3 Doctoral Program in Biomedical Sciences and Public Health, International Doctorate Program, National University of Distance Education (UNED), Madrid, 28015 Spain; 4 Puertollano Integrated Care Management. Health Service of Castilla-La Mancha, Unit of Teaching and Research, Santa Bárbara Hospital, Castilla-La Mancha, Spain; 5 Communication, Food and Consumption Research Group (FOODCO), Alicante, Spain

**Keywords:** Food advertising, Marketing, Childhood obesity, Television, Socio-economic inequalities

## Abstract

**Background:**

The influence of food advertising on food preferences and consumption could also contribute to the socio-economic inequalities among Spanish children in terms of eating habits and childhood obesity. Although the main food advertising channel targeted at children in Spain is television, available studies estimate exposure indirectly by combining content data with audience data. The aim of this study was therefore to describe the frequency of exposure to television advertising of unhealthy foods and drinks, measured directly, among Spanish children and adolescents, and analyse its socio-economic inequalities.

**Methods:**

Observational study of television advertising impacts in a sample of 1590 children aged 4 to 16 years drawn from a consumer panel representative of the Spanish population in this age group, over the course of a full week of broadcasting in February 2022. The sample was obtained through stratified random sampling by Autonomous Region, with quotas being set by reference to socio-demographic variables. Exposure was measured with an audiometer, and the nutrient content of the food and drink advertised was analysed using the nutrient profile of the WHO Regional Office for Europe. We used the Chi-squared test to analyse possible differences in advertising coverage by socio-economic level.

**Results:**

The participants saw a weekly mean of 82.4 food and drink commercials, 67.4 of which were for unhealthy products (81.8%), mostly outside the child-protection time slot. On average, low-social class participants received 94.4% more impacts from unhealthy food and drink advertising than did high-class participants (99.9 vs. 51.4 respectively). The mean advertising coverage of unhealthy foods and drinks was 71.6% higher in low-class than in high-class participants (10.9% vs. 18.7%; *p* = 0.01).

**Conclusion:**

Spanish children and adolescents received an average of 10 impacts per day from television spots for unhealthy foods and drinks. The exposure of low-class children is double that of high-class children, a finding compatible with the high prevalence of childhood obesity in Spain and the related socio-economic inequalities. To protect Spanish minors from the harmful effects of food advertising and reduce the related social health inequalities would require the implementation of a 24:00 watershed for unhealthy food advertising on television.

**Supplementary Information:**

The online version contains supplementary material available at 10.1186/s12889-023-17410-z.

## Background

Recent decades have witnessed a progressive abandonment of the Mediterranean diet among children and adolescents in Spain [1], and a parallel replacement of fresh produce by ultraprocessed food and drinks in the Spanish householder’s market basket [2]. Whereas only 37.1% of children and 34.7% of adolescents consume fruit daily [3, 4], a recent study observed a mean intake of 8 ultraprocessed portions per day in adolescents [5], a level of consumption related with a relative increase of 62% in all-cause mortality in the Spanish adult population [6]. The situation is more serious for children and adolescents of parents with a low educational level or low family income, who display worse adherence to the Mediterranean diet, with a lower consumption of olive oil, fruit, vegetables, legumes and fish [3, 4].

In all commercial channels, and eminently in television (TV), food advertising is frequent and persuasive, and most of the products advertised are unhealthy [7]. Such marketing is one of the main causes of the shift in children’s eating patterns because it influences their food preferences, buying habits -including their demands on parents- and food consumption [7]. This influence is both resistant, in that children continue to choose the advertised products more frequently even when they are advised by their parents to choose healthier products [8], and persistent, in that time of exposure to TV food advertising in adolescents is a predictor of worse eating habits over the next five years [9].

In Spain, the main food advertising channel targeted at children is TV, with 80.4% of total industry expenditure devoted to this item in 2018 [10]. Although the PAOS Code of self-regulation of food advertising directed at children under the age of 12 years along with a food and drink reformulation plan have been implemented in Spain in the last decade, based on voluntary agreements between the Government and the food industry, from 2012 to 2020 close on one in four TV commercials were for food and drinks, 64–75% of which were unhealthy products, high in fat, sugar, salt and calories [11, 12].

In addition to content analysis (potential exposure), there are two methods for monitoring children’s exposure to TV food advertising: estimation of exposure, by pooling content data with audience data; and direct measurement of exposure [13]. A 2012 study which combined advertising content and child audience data in Spain to estimate exposure to TV food advertising, calculated that children aged 4 to 12 years received a daily mean of 12 impacts from commercials for products high in calories, saturated fats, sugar and salt [11]. Advertising impact is the measure of a commercial’s viewing by an individual.To our knowledge, there is no study that has directly measured Spanish children’s frequency of exposure to TV advertising of unhealthy foods and drinks, or the influence of socio-economic level on such exposure. In other countries, most studies that have measured children’s exposure to unhealthy food advertising have observed a higher frequency of exposure in ethnic minority groups and those of a low socio-economic level [14].

The aim of this study was thus to describe the frequency of exposure to TV advertising of unhealthy foods and drinks, measured directly, among Spanish children and adolescents aged 4 to 16 years, and analyse its socio-economic inequalities.

## Methods

### Study design and subjects

We conducted an observational cross-sectional study based on continuous measurement of TV advertising impacts received by a sample of 1590 children and adolescents aged 4 to 16 years, representative of the Spanish population in this age group, over the course of a full week of broadcasting (21 to 27 February 2022). Advertising impact is the measure of a commercial’s viewing. An individual may view the same commercial broadcast at different times or TV channels, each viewing representing an advertising impact. Total advertising impacts are the sum of commercial’s viewing of all individuals during the study period. The sample was drawn from a consumer panel made up of 5720 households which use audiometers to record TV viewing by all the members. Households serving on the panel undergo an annual rotation of sufficient proportions to ensure that their maximum duration in the sample never exceeds 7 years. At all events, the annual rotation is never less than 14%.

The sample was obtained through stratified random sampling by Autonomous Region (*Comunidad Autónoma*). The sample was aproportional to achieve a sample size larger than that which would otherwise correspond to the smallest Autonomous Regions. Sampling points were selected on the basis of the census sections of the National Statistics Institute. Sample sections were chosen with a probability proportional to their size, so that all households would be equiprobable. To obtain the greatest sample dispersion possible in each census section, we allowed for no more than 1 panellist household. Similarly, provided that there was a sufficient number of towns for the region/habitat intersections, the target for the setting of sample quotas, this ensured that there would be no more than 1 panellist household per town. Households in each section were selected randomly, with quotas being set to ensure proportions similar to those of the target population, in terms of sex, age, number of persons in the home, habitat, social class, language, housewife’s activity, number of TV sets, possession of a remote-control device, video or DVD, number of TV channels received in the home, and number of homes with TDT reception or subscribed to digital or cable TV platforms.

### Data-collection and study variables

To measure the number and profile of the viewers who are watching TV during the course of any given day, the households belonging to the consumer panel use an audiometer. This is a remote-control device, similar in appearance to a TV remote, which registers the TV programme being watched at any given time and the person or persons in the household who are watching it, by means of a precoded key for each household member present in front of the TV set.

The consumer panel provides aggregate information on the number of total advertising impacts received from the food sector and each food or drink advertised on TV channels having an audience share of the target population (boys and girls aged 4 to 16 years) of over 1.5% (Telecinco, Antena 3, Cuatro, FDF, NEOX, La Sexta, NOVA, Boing and Disney Channel), by age strata (4–10 and 11–16 years) and three categories of socio-economic level by tertiles of household income. The indicator of a given household’s socio-economic level was obtained on the basis of the following variables: main wage-earner’s educational level, profession and activity (gainfully employed, retired, unemployed or economically inactive); size of household; and number of individuals having an income [15]. A factorial analysis using mean national incomes by occupation found those four variables explained more than 85% of the variance of the household income. Then multiple regression with the four variables was used to estimate household income of participants. The panel also furnished data on total food-sector advertisements and impacts, by time slot (02:30 − 07:00, 07:00–14:00, 14:00–17:00, 17:00–20:30, 20:30 − 24:00 and 24:00–02:30) and TV channel.

#### Nutritional information about advertised products

The nutritional composition of the food and drink advertised was obtained from the websites of the manufacturing companies or from the online platforms of product retail chains. Where a product’s nutritional information could not be located on the Internet, its label was consulted after the item had been purchased at a food store or supermarket. In any case where all or part of the nutritional information could not be obtained by any of these means, we consulted the Spanish food nutritional composition database [16].

The nutrient profile of the food and drink advertised was analysed using the nutrient profile of the WHO Regional Office for Europe, which classifies it into 17 food categories [17]. According to the category to which a product belongs and its nutritional composition, the system determines whether it is considered healthy or unhealthy and, by extension, whether its advertising would or would not be permitted. In 5 categories no product is considered healthy and, therefore, none could be advertised: chocolates and sugar confectionery, pastry and cookies, juices, energy drinks, and edible ices. In the remaining cateogories, products are only considered healthy when they do not exceed certain thresholds established for the amounts of sugars, fats, fats saturated, salt, sweeteners or energy. The thresholds are specific for each food category. For example, breaskfat cereals could be advertised if they don’t exceed 10 g of total fat, 15 g of total sugars, and 1.6 g of salt por 100 g.

### Statistical analysis

Based on data of the number of commercials and impacts, we performed a descriptive analysis of the exposure indicators: percentage of unhealthy food and drink advertisements and their impact on total food-sector advertising and impacts, on the one hand, and on total advertisements and impacts of all advertising sectors, on the other. The indicators relating to advertisements were also calculated in subgroups defined by TV channels and broadcasting timetables. Furthermore, we calculated mean impacts by age and socio-economic subgroups. In addition to the number of participants and impacts, Kantar also provided data on commercials’ coverage (percentage of participants impacted at least once by a commercial of a particular product) by age and socio-economic level. Hence, we used the non-parametric Chi-squared test to analyse possible differences in advertising coverage by age or socio-economic level. All analyses were performed using the STATA statistical software programme [18].

## Results

Table 1 shows the socio-economic characteristics of the sample: 47.4% were girls and 52.6% were boys aged 4 to 10 years. Most of the participants were middle-class (40.6%) and 17% were of a low social class. The socio-demographic distribution was very similar to that of the Spanish population aged 4 to 16 years, though the percentage of males in the sample was slightly higher (52.6% vs. 51.4%), that of adolescents slightly lower (47.4% vs. 49.4%), and that of low-social class participants slightly higher (17% vs. 15.9%).

**Table 1:** Socio-demographic characteristics of the study sample, representative of the Spanish population aged 4–16 years: 2022

	n	%	% population
**Sex**			
**Male**	836	52.6	51.4
**Female**	754	47.4	48.6
**Total**	1590	100.0	
**Age in years**			
**4 to 10**	837	52.6	50.6
**11 to 16**	753	47.4	49.4
**Total**	1590	100.0	
**Social class**			
**Low**	270	17.0	15.9
**Middle**	646	40.6	40.1
**High**	674	42.4	44.1
**Total**	1590	100.0	

Table 2 shows food-sector advertising spots and impacts by broadcasting time slot and TV channel: 18.5% of all advertising spots and 19.2% of all advertising impacts involved food and drink commercials. The highest percentage of food-sector advertising spots and impacts occurred in the 7:00 to 14:00 time slot, with 21.6% and 23.1% of the total respectively. By TV channel, Boing ranked first in food-sector advertising spots and impacts, with 26.8% and 29.4% of the total respectively.

**Table 2:** Food-sector advertising spots and impacts, by time slot and TV channel, in the Spanish population aged 4 to 16 years: 2022

**Broadcasting time slot**	**Spots**	**%**	**% of total campaigns**	**Impacts**	**%**	**% of total campaigns**
02:30 − 07:00	9	0.1	1.3	8	0.0	0.6
07:00–14:00	2117	32.9	21.6	18,468	14.1	23.1
14:00–17:00	1194	18.5	18.2	31,238	23.8	17.8
17:00–20:30	1385	21.5	20.8	32,764	25.0	22.5
20:30 − 24:00	1357	21.1	17.1	43,424	33.1	17.4
24:00–02:30	375	5.8	12.1	5179	4.0	16.2
**Channel**	**Spots**	**%**	**% of total campaigns**	**Impacts**	**%**	**% of total campaigns**
T5	741	11.5	18.4	26,915	20.5	18.7
A3	724	11.2	18.8	20,978	16.0	18.0
CUATRO	578	9.0	15.0	8886	6.8	14.6
FDF	616	9.6	17.2	9064	6.9	16.9
NEOX	1301	20.2	25.3	10,587	8.1	21.7
LA SEXTA	538	8.4	14.3	7278	5.6	14.7
NOVA	856	13.3	20.4	7598	5.8	22.0
BOING	850	13.2	26.8	35,910	27.4	29.4
DISNEY CHANNEL	233	3.6	7.5	3865	2.9	7.2
**Total**	6437	100.0	18.5	131,081	100.0	19.2

Most of the food and drink commercials were broadcast during the 7:00–14:00 time slot, with 32.9% of the total, followed by the 17:00–20:30 time slot, with 21.5%. In contrast, the greatest part of all food and drink advertising impacts occurred during the 20:30 − 24:00 time slot, with 33.1% of the total, followed by the 17–20:30 slot, with 25%.

The majority of food and drink advertising spots were broadcast on the NEOX channel, with 20.2% of the total. Even so, the greatest part of the impact of food and drink advertising was generated by the BOING channel, with 27.4%, and the T5 channel, with 20.5%.

Table 3 shows the mean impacts of food and drink advertising by age group and social class. During the week for which data were recorded, the participants saw a mean of 82.4 food and drink commercials, 67.4 of which were for unhealthy products (81.8%). The frequency of impacts of unhealthy food and drink advertising was higher in the 4 to 10 year age group, with a mean of 69.3, than in the 11–16 age group, with a mean of 65.4. On average, low-social class participants received 41.7% more impacts from unhealthy food and drink advertising than did the middle-class participants, and 94.4% more than the high-class participants (99.9 vs. 70.5 and 51.4 respectively).

**Table 3:** Number of and mean impacts per week of food and drink advertising, by age and social class, in the Spanish population aged 4 to 16 years: 2022

	Total		Age (years)				Social class					
			4–10		11–16		High		Middle		Low	
Nutrient profile	No.	Mean/week	No.	Mean	No.	Mean	No.	Mean	No.	Mean	No.	Mean
Unhealthy	107,191	67.4	57,969	69.3	49,223	65.4	34,644	51.4	45,555	70.5	26,984	99.9
Healthy	15,026	9.5	7493	9	7532	10	4958	7.4	6356	9.8	3711	13.7
Not applicable	8868	5.5	4005	4.8	4862	6.5	3015	4.5	3704	5.7	2148	8
**Total**	131,081	82.4	69,463	83	61,618	81.8	42,619	63.2	55,616	86.1	32,846	121.7

During the week of study, different unhealthy food and drink advertisements were broadcast for 89 foods and drinks, 62 of which were unhealthy (72.9%). Table 4 shows the advertising coverage of the food sector as a whole and of the 14 unhealthy foods and drinks that attained coverages of more than 15%. The nutritional information of these products by food categories of the nutritional profile is provided in a supplementary file. Overall coverage of the food sector was 59.7% and showed differences by social class, going from 55.8% in high-class to 68% in low-class participants (*p* < 0.01). The 3 products with highest coverages were Cola Cao, Babybel mini cheese portions and Actimel liquid yoghurt, with 30.4%, 24.6% and 23.6% respectively. Whereas coverage of Babybel mini cheese portions was higher in the 4–10 than in the 11–16 year age group (32.5% vs. 16.4%; *p* < 0.01), coverage of Actimel liquid yoghurt was higher in the 11–16 than in the 4–10 year age group (26.5% vs. 20.9%; *p* = 0.01). Coverage was higher in low-class participants for all foods and drinks in the table, with the most marked differences between high and low social classes being for Cuétara Choco Flakes cereals (15.2% vs. 32.6%; *p* < 0.01) and Cuétara María biscuits (13.6% vs. 27.3%; *p* < 0.01). The mean advertising coverage of unhealthy foods and drinks was 71.6% higher in low-class than in high-class participants (10.9% vs. 18.7%; *p* = 0.01).

**Table 4:** Coverage of unhealthy food and drink advertising in the Spanish population aged 4 to 16 years: 2022

	Coverage (%)							
	Total	Age (years)			Social class			
Product		4–10	11–16	*p*	High	Middle	Low	*P*
**El Pozo processed meat**	20.3	16.7	24.0	< 0.01	19.7	19.1	25.4	0.07
**Campofrío sliced ham**	16.3	13	19.6	< 0.01	14.2	15.2	24.5	< 0.01
**Cuétara Cereals Choco Flakes**	21.4	26.5	16.3	< 0.01	15.2	23.7	32.6	< 0.01
**Kinder Bueno chocolate bars**	18.7	15.6	21.9	< 0.01	15.5	18.8	27.1	< 0.01
**Valor chocolates**	17.5	14.6	20.4	< 0.01	14.8	17.7	24.4	< 0.01
**CUETARA/TOSTA RICA/OCEANIX/BISCUITS**	18.2	24.3	12.0	< 0.01	14.9	19.1	25.2	< 0.01
**Chocolate wafers**	23.1	19.4	26.8	< 0.01	20.5	22.4	31.7	< 0.01
**Cuétara /Tosta Rica María biscuits**	18.6	24.6	12.5	< 0.01	13.6	20.7	27.3	< 0.01
**Artiach /Dinosaurus biscuits /Brownie cupcakes**	21.6	23.3	19.9	0.10	15.9	24.0	31.4	< 0.01
**Old el Paso Mexican tortillas.**	19.6	17.6	21.7	0.04	17.2	19.7	26.0	0.01
**Cola Cao**	30.4	31.8	28.9	0.22	24.4	31.4	42.8	< 0.01
**García Baquero semi-cured cheese**	20.1	19.6	20.5	0.67	17.1	21.2	25.8	0.01
**Mini Babybel mini cheese portions**	24.6	32.5	16.4	< 0.01	20.3	25.7	33.2	< 0.01
**Danone Actimel liquid yogurt**	23.6	20.9	26.5	0.01	19.9	24.6	31.3	< 0.01
Mean*	13.3	12.8	13.8	0.55	10.9	13.6	18.7	0.01
**Total**	59.7	61	58.2	0.75	55.8	60.6	68.0	< 0.01

* Mean coverage of unhealthy food and drink commercials, including those not shown in the table

## Discussion

In Spain, a fifth of all advertisements broadcast by TV were for the food sector. Although most of the spots in this sector were screened during the 7:00–14:00 time slot and the channel with most advertising spots was Neox, targeted at a juvenile public, the greatest part of the advertising impact on the children who participated in the study was generated by children’s (Boing) and generalist channels alike (Telecinco), during the 20:30 − 24:00 time slot, outside the child-protection timetable. Over 80% of the impact was for unhealthy foods and drinks, with a weekly mean of 67.4 impacts. The mean advertising coverage of unhealthy food and drink commercials and the weekly mean impacts received were 71.6% and 94.4% higher respectively in low-class than in high-class children and adolescents.

With 18.5% of advertising spots, a figure somewhat lower than that of previous studies [11, 19], food continues to be one of the leading TV advertising sectors in Spain. Although the percentage of unhealthy foods and drinks advertised (72.9%) was very similar to that observed in 2017 and 2020 [12], the percentage of their advertising impacts exceeded these figures, accounting for 81.8% of the total food-sector impact. The daily mean impact of unhealthy foods and drinks observed in our study was 9.6, somewhat lower than the figure estimated in a previous 2012 study [11], yet twice as high as the European Union mean in the same age range, which stands at 4.7 [20], and around three times higher than that reported by a recent study in Canada [21].

All studies which have analysed the relationship between socio-economic class and exposure to TV advertising of unhealthy foods and drinks have reported negative associations with parents’ education, occupation and household income [14], the three variables used in this study to obtain the indicator of socio-economic level. In one of the studies, based on audience data from a consumer panel in the United Kingdom, exposure to unhealthy food advertising among lower-income spectators was slightly more than double that among higher-income spectators [22], a very similar association to that observed by our study (2.1 vs. 1.9 times higher). This negative socio-economic gradient has also been observed for exposure to advertising on hoardings and in public transport [14, 23], but in the case of online advertising there is little information and the data are non-concordant [24, 25]. In a study conducted in Spain in 2022 targeting children aged 8 to 16 years, the frequency of exposure to unhealthy food and drink advertising among school-goers in lower mean income areas was not even as much as 10% higher than that of schoolchildren from higher mean income areas [26], a socio-economic gradient of a much lower magnitude than that seen in our study. These differences may be due to the way of measuring exposure because, whereas our data were obtained by direct measurement, those of the PASOS study were self-reported. Among Canadian adolescents who self-reported their level of advertising exposure, the differences in frequency of exposure by socio-economic level were of scant magnitude, somewhere in the region of 4% [25]. In another study which used self-reported data on adults in the United Kingdom, the socio-economic gradient observed was of a lower magnitude than that observed with direct measurement: 1.4 vs. 2.1 times higher exposure to unhealthy food and drink advertising in a population of a low socio-economic level [22, 24]. Self-reported data are susceptible to information biases that limit their reliability and accuracy, and even more so when the questionnaires applied have not been previously validated, biases that could be greater still in the child and adolescent population, since children are unable to identify advertising until they reach the 6–12 year age range and are not aware of its persuasive intention and tactics used until late adolescence [27, 28].

Our results are compatible with TV viewing data for Spanish children and adolescents. Although most of the food and drink advertising spots were broadcast during the 7:00 to 14:00 time slot, most of the advertising impacts were generated from 20:30 to 24:00, the peak child-audience viewing time in Spain [29]. Similarly, while the Neox channel ranked first in terms of advertising spots, the Boing and Telecinco channels headed the ratings in terms of impact because their audience is higher when it comes to children and adolescents [29]. The higher number of impacts observed for children of a low socio-economic class is also compatible with the TV viewing gradient for Spanish children and adolescents by social class and parental educational level or occupation [30–32]. Exposure to TV food advertising, under both experimental and real-life conditions, increases the unhealthy food and drink consumption and caloric intake of children and adolescents alike [33, 34]. Several recent studies have observed that these effects of food advertising, either do not change according to socio-economic level [35, 36], or are of a higher magnitude in persons of a low socio-economic level, who would be more susceptible to the pernicious influence of food advertising [37]. In the COSI study on 6 to 9-year-old children from countries in the WHO European Region, the association between objective measures of socio-economic level and consumption of sugar-sweetened drinks was of a higher magnitude in Spain than in other countries, especially when it came to parents’ educational level and occupation: the percentage of children who consumed more than 3 sugar-sweetened drinks per week was double among children of parents with low- versus high-level jobs [38], a finding compatible with the higher exposure of Spanish children to this type of advertising observed in this study, as compared to other European countries [20]. Hence, although high advertising exposure does not mean high consumption of unhealthy food, it might be that the worse eating habits of Spanish children and adolescents of a low socio-economic class, characterised by a lower intake of fruit and vegetables and higher intake of ultraprocessed foods and sugar-sweetened drinks [3, 4, 39], could, in certain measure, be due to their greater exposure to TV advertising of unhealthy foods and drinks, which would, in turn, contribute, on the one hand, to the persistence of great social inequality in childhood obesity figures in Spain [39, 40], and, on the other, to the fact that Spain is a country with one of the highest prevalences of overweight and obesity in Europe [41, 42]. Hovewer, obesity is a more complex phenomenon, with many factors affecting it, including sedentarism and lack of exercise that are also more prevalent in people from low economic status [39, 40]. Hence, coping obesity requires a holistic approach, including interventions at the policy and community level to improve economic and cultural resources in low-income environments.

Spanish children and adolescents’ high exposure to unhealthy food and drink advertising observed by our study is accounted for by the absence of regulation of the matter in Spain, since the PAOS Code of self-regulation of food advertising directed at children under the age of 12 years, which is of a voluntary nature, regulates the marketing power, but neither the nutrient profile of the products advertised nor the broadcasting frequency of commercials [43]. Recently, the Spanish Ministry of Consumer Affairs drew up a proposed statutory regulation to govern this type of advertising, which has not prospered due to the opposition of the food and advertising industries, with the support of the Ministry of Agriculture, stalling tactics which have been denounced by a number of civil society organisations that have spent years demanding that this sphere be regulated, in order to protect the fundamental rights of Spanish children [44]. The Audiovisual Communication Act (*Ley de Comunicación Audiovisual*) urges the Government to impose statutory restrictions on the advertising of unhealthy foods and drinks in cases where codes of conduct are not effective enough to reduce minors’ exposure to such advertising [45], something that has not only been borne out by this study, but also corroborates the findings of previous studies [11, 12]. In light of our results, to be effective in terms of television, any future regulation in Spain would have to cover those areas where minors experience their greatest exposure to unhealthy food and drink advertising, by covering the 20:30 to 24:00 time slot on all television channels, including the generalist channels, especially when the above Act has relaxed the criteria governing the distribution of time devoted to advertising, allowing a higher concentration of commercials during peak child-audience viewing times. Indeed, the application of this type of regulation in Chile served in great measure to reduce exposure to unhealthy food and drink advertising, with this reduction being pronounced in children who spent more time watching TV [46], thereby demonstrating that it is an effective measure, not only for protecting children from exposure, but also for reducing the social inequalities in the pernicious health effects of such advertising.

### Limitations

Although this is the first study to analyse socio-economic inequalities in exposure to TV advertising of unhealthy foods and drinks, measured directly in a representative sample of the Spanish child and adolescent population, it also suffers from some limitations. Firstly, sampling, rather than being completely random, was instead aproportional in order to over-represent the smallest Autonomous Regions, with a maximum of 1 household per census section or town to increase sample dispersion, and with quotas being set according to socio-demographic variables in the selection of households to ensure proportions similar to those of the target population. Secondly, the study was confined to one of the many commercial channels to which children and adolescents are exposed [47]. Moreover, not all TV channels that are broadcast in Spain were included, with the result that the level of exposure to TV food advertising is underestimated. That said, however, the channels that were not included enjoy an audience share of the target population of under 1.5% [29], so that any bias could be expected to be of a small magnitude. Thirdly, our study did not record exposure to online TV food advertising or food advertising deriving from TV advertising strategies other than those of a traditional commercial kind, such as product placement or programme sponsorship, something that would again contribute to underestimate exposure, though such bias would likewise be expected to be of a small magnitude and non-differential.

## Conclusions

This study shows the high level of exposure of Spanish children and adolescents to TV advertising of unhealthy foods and drinks, with at least 10 impacts per day of this type of commercial, mostly outside the child-protection timetable. Furthermore, major socio-economic inequalities are in evidence, since the exposure of low-class children is double that of high-class children. To protect Spanish minors from the harmful effects of TV food advertising and reduce the related social health inequalities, a effective regulation would require the implementation of a 24:00 watershed for unhealthy food advertising on TV.

### Electronic supplementary material

Below is the link to the electronic supplementary material.

Supplementary Material 1

## Data Availability

The datasets used and analysed during the current study are available from the corresponding author on reasonable request.

## Abbreviations

TV: television
WHO: World Health Organisation
