# Supplementary material for: Differences in children’s exposure to television advertising of unhealthy foods and beverages in Spain by socio-economic level
Source: BMC Public Health. 2024 Mar 7;24:739. doi: 10.1186/s12889-023-17410-z (PMC10921598; doi:10.1186/s12889-023-17410-z)
Supplement: Supplementary file 1 — Supplementary Material 1 [file 12889_2023_17410_MOESM1_ESM.docx]

| **Nutritional information of the food and drink to which Spanish children 4 to 16 years are exposed according to the criteria of the WHO Regional Office for Europe nutrient profile model** | | | | | | | |  |  |
| --- | --- | --- | --- | --- | --- | --- | --- | --- | --- |
|  |  | **Marketing not permitted if product exceeds, per 100 g** | | | | | | | |
| **FOOD PRODUCT** | **food category** | **kcal** | **kjul** | **total fat (g)** | **sat. fat(g)** | **total sugars (g)** | **added sugars (g)** | **proteins (g)** | **salt (g)** |
| El Pozo processed meat | Processed meat, poultry, fish and similar | 388 | 1608 | **29** | 10 | 5 | 4,5 | 26,5 | **4** |
| Campofrío sliced ham | Processed meat, poultry, fish and similar | 86 | 362 | 1,5 | 0,5 | 3 | 3 | 15 | **2,1** |
| Cuétara Cereals Choco Flakes | Breakfast cereals | 450 | 1894 | **14** | 4 | **73** | 26 | 6,3 | 0,53 |
| Kinder Bueno chocolate bars | Chocolate and sugar confectionery, energy bars, and sweet toppings and desserts | **Marketing not permitted** | | | | | | | |
| Valor chocolates | Chocolate and sugar confectionery, energy bars, and sweet toppings and desserts | **Marketing not permitted** | | | | | | | |
| CUETARA/TOSTA RICA/OCEANIX/BISCUITS | Cakes, sweet biscuits and pastries; other sweet bakery wares, and dry mixes for making such | **Marketing not permitted** | | | | | | | |
| Chocolate wafers | Cakes, sweet biscuits and pastries; other sweet bakery wares, and dry mixes for making such | **Marketing not permitted** | | | | | | | |
| Cuétara /Tosta Rica María biscuits | Cakes, sweet biscuits and pastries; other sweet bakery wares, and dry mixes for making such | **Marketing not permitted** | | | | | | | |
| Artiach /Dinosaurus biscuits /Brownie cupcakes | Cakes, sweet biscuits and pastries; other sweet bakery wares, and dry mixes for making such | **Marketing not permitted** | | | | | | | |
| Old el Paso Mexican tortillas. | Ready-made and convenience foods and composite dishes | **237** | 996 | 7 | 0,9 | **35,6** | 4,5 | 6,5 | **2,08** |
| Cola Cao | Chocolate and sugar confectionery, energy bars, and sweet toppings and desserts | **Marketing not permitted** | | | | | | | |
| García Baquero semi-cured cheese | Cheese | 384,4 | 1593,7 | **32** | 21,6 | 1,6 | 1,6 | 22,5 | **1,5** |
| Mini Babybel mini cheese portions | Cheese | 308 | 1280 | **24** | 16 | 0,5 | 0,5 | 23 | **1,8** |
| Danone Actimel liquid yogurt | Yoghurts, sour milk, cream and other similar foods | 73 | 307 | 1,6 | 1,1 | **10,8** | 3 | 0,1 | 0 |

Bold type means that the established tresholds for the amounts of sugars, fats, fats, saturated, salt, sweeteners or energy in each food category have been exceed according to the WHO Regional Office for Europe’s Nutrient Profile Model of 2015. Available from: http://apps.who.int/iris/bitstream/handle/10665/152779/Nutrient%20Profile%20Model.pdf;jsessionid=50BEF10AFF7AA509B55AA6819DA455C0?sequence=1
